# Increased *HSP70* and *TLR2* Gene Expression and Association of *HSP70* rs6457452 Single Nucleotide Polymorphism with the Risk of Chronic Obstructive Pulmonary Disease in the Croatian Population

**DOI:** 10.3390/diagnostics11081412

**Published:** 2021-08-04

**Authors:** Iva Hlapčić, Marija Grdić Rajković, Andrea Čeri, Sanja Dabelić, Sanja Popović-Grle, Margareta Radić Antolic, Jelena Knežević, Asta Försti, Lada Rumora

**Affiliations:** 1 Department of Medical Biochemistry and Haematology, Faculty of Pharmacy and Biochemistry, University of Zagreb, 10000 Zagreb, Croatia; iva.hlapcic@pharma.unizg.hr (I.H.); mgrdic@pharma.hr (M.G.R.); andrea.ceri@pharma.unizg.hr (A.Č.); 2 Department of Biochemistry and Molecular Biology, Faculty of Pharmacy and Biochemistry, University of Zagreb, 10000 Zagreb, Croatia; sanja.dabelic@pharma.unizg.hr; 3 Clinical Department for Lung Diseases Jordanovac, University Hospital Centre Zagreb, 10000 Zagreb, Croatia; spopovi1@kbc-zagreb.hr; 4 School of Medicine, University of Zagreb, 10000 Zagreb, Croatia; 5 Clinical Institute of Laboratory Diagnostics, University Hospital Centre Zagreb, 10000 Zagreb, Croatia; mradicantolic@gmail.com; 6 Ruđer Bošković Institute, Division of Molecular Medicine, Laboratory for Advanced Genomics, 10000 Zagreb, Croatia; Jelena.Knezevic@irb.hr; 7 Department of Integrative Medicine, Faculty for Dental Medicine and Health, Josip Juraj Strossmayer University of Osijek, 31000 Osijek, Croatia; 8 German Cancer Research Center (DKFZ), German Cancer Consortium (DKTK), Division of Pediatric Neuroon-Cology, 69120 Heidelberg, Germany; a.foersti@kitz-heidelberg.de; 9 Hopp Children’s Cancer Center (KiTZ), 69120 Heidelberg, Germany

**Keywords:** chronic obstructive pulmonary disease, heat shock protein 70, toll-like receptor 2, toll-like receptor 4, single nucleotide polymorphism

## Abstract

Heat shock protein 70 (Hsp70) engages Toll-like receptors (TLR) 2 and 4 when found in the extracellular compartment and contributes to inflammation in chronic obstructive pulmonary disease (COPD). Since there is growing evidence for the genetic risk factors for COPD, the gene expression of *HSP70*, *TLR2* and *TLR4* was determined, as well as the association between *HSP70*, *TLR2* and *TLR4* single nucleotide polymorphisms, (SNPs) and COPD. The gene expression was assessed in peripheral blood cells of 137 COPD patients and 95 controls by a quantitative polymerase chain reaction (qPCR), while a total of nine SNPs were genotyped by TaqMan allelic discrimination real-time PCR. *HSP70* and *TLR2* gene expression was increased in COPD patients compared to the controls, regardless of the disease severity and smoking status of participants. The rs6457452 SNP of *HSP70* was associated with COPD, indicating the protective role of the T allele (OR = 0.46, 95% CI = 0.24–0.89, *p* = 0.022). Furthermore, COPD C/T heterozygotes showed a decreased *HSP70* mRNA level compared to COPD C/C homozygotes. In conclusion, *HSP70* and *TLR2* may have a role in the pathogenesis of COPD, and the *HSP70* rs6457452 variant might influence the genetic susceptibility to COPD in the Croatian population.

## 1. Introduction

Chronic obstructive pulmonary disease (COPD) is a multicomponent heterogeneous inflammatory disease characterized by persistent respiratory symptoms and airflow limitation [1]. It is known that the development of COPD is influenced by several environmental and genetic factors. Tobacco exposure has been recognized as the main environmental risk factor for COPD [2], yet only 10–20% of smokers develop airway obstruction [3]. COPD has both pulmonary and extrapulmonary manifestations, and systemic chronic inflammation has been identified as a potential COPD endotype [4]. It is considered that the persistent low-grade inflammation in stable COPD might be caused by increased levels of many inflammatory mediators [5], reactive oxygen and nitrogen species [6], and pathogen-associated molecular patterns or damage-associated molecular patterns (DAMPs) such as extracellular heat shock protein 70 (eHsp70) [7,8,9]. eHsp70 is thought to activate proinflammatory responses by binding to Toll-like receptors (TLRs) 2 and 4, leading to increased cytokines concentrations [10].

Hsp70 is predominantly an intracellular protein involved in the maintenance of protein homeostasis, especially in the presence of stressors such as heat, bacterial, and viral infections [11]. However, the role of Hsp70 in COPD pathogenesis remains to be elucidated. Increased gene expression of *HSP70* and its protein concentration was observed in lung tissues of COPD patients, and *HSP70* mRNA levels were negatively correlated with lung function. Moreover, *HSP70* expression in lung tissues was associated with airway obstruction severity and smoking status [12]. In the study by Ambrocio-Ortiz et al., expression of *HSP70* was examined in sputum, and they found a decrease in the COPD smoking group compared to controls who were mostly smokers [13].

Considering TLR2 and TLR4, they are expressed by a variety of cells involved in innate and adaptive immune responses. Cigarette smoke and Hsps can affect TLR2 and TLR4 protein concentration and the mRNA level in the respiratory tract. Our previous study showed that cigarette smoke or eHsp70 can inhibit the expression of *TLR2* and *TLR4* in bronchial epithelial cells in COPD [14]. Moreover, *TLR2* and *TLR4* expression was decreased in the lung parenchyma of COPD patients with severe disease, while the gene expression of both receptors was increased in the airway epithelium of COPD patients in mild and moderate stages [15]. However, when *TLR2* and *TLR4* gene expression was determined in alveolar macrophages from the bronchoalveolar lavage of COPD smokers, there was no difference in the mRNA levels compared to both the control of non-smokers and control of smokers [16].

In general, the gene expression could differ in various types of samples used in the analysis. Sputum reflects the central airways, while bronchoalveolar lavage represents the peripheral airways [17]. On the other hand, blood is an easily available sample that is representative of systemic compartment, which is also important in COPD. To the best of our knowledge, this is the first study in which blood samples were used to assess the basal *HSP70*, *TLR2* and *TLR4* gene expression in stable COPD without any further in vitro treatments or stimulations.

COPD is a complex disease with a genetic background. Single nucleotide polymorphisms (SNPs) may be associated with changes in gene expression, affect gene product and thus contribute to susceptibility to disease. *HSP70* polymorphisms have been found to be related to various diseases such as pulmonary fibrosis [18,19], atherosclerosis [20], coronary heart disease [21] and COPD [13]. Furthermore, the *HSP70* rs1061581 variant was associated with COPD in the Croatian population [22]. Genetic polymorphisms in *TLR2* and *TLR4* genes were also significantly related to altered risk of COPD [23,24,25] as well as to reduced lung function and an increased number of inflammatory cells in the sputum of COPD patients [24].

The aim of this study was to investigate the gene expression of *HSP70*, *TLR2* and *TLR4* in the peripheral blood cells of COPD patients and to explore whether there is an association between the gene expression and disease severity, and smoking. Genotype and allele distribution of selected *HSP70*, *TLR2* and *TLR4* polymorphisms were determined in healthy subjects and COPD patients, and a potential association between SNPs and COPD was assessed. Finally, we explored the relation between *HSP70*, *TLR2* and *TLR4* mRNA levels, and investigated polymorphisms.

## 2. Materials and Methods

### 2.1. Study Population

The retrospective study comprised of 137 COPD patients in the stable phase of the disease, and 95 controls. Stable COPD was defined as no exacerbations during at least three physician visits in the previous 3 months, with no changes in respiratory therapy and no symptoms of a lower respiratory tract infection. All participants were recruited at the Clinical Department for Lung Diseases Jordanovac, University Hospital Centre Zagreb (Zagreb, Croatia) after they agreed to participate voluntarily in the study and signed an informed consent form. The study was approved by the Ethical Committee of the University Hospital Centre Zagreb and the Ethical Committee for Experimentation of the University of Zagreb Faculty of Pharmacy and Biochemistry (Zagreb, Croatia) (approval protocol numbers: 02/21/JG on 29 August 2014 and 251-62-03-14-78 on 10 September 2014, respectively).

The diagnosis of COPD was made according to the guidelines [1], after measuring the spirometry parameters—forced expiratory volume in the first second (FEV_1_) and forced vital capacity (FVC). If FEV_1_/FVC was under the value of 0.70, airway obstruction was confirmed. COPD patients were classified into GOLD stages based on FEV_1_ as follows: GOLD 1 (FEV_1_ ≥ 80%) (*n* = 0), GOLD 2 (50% ≤ FEV_1_ < 80%) (*n* = 47), GOLD 3 (30% ≤ FEV_1_ < 50%) (*n* = 50) and GOLD 4 (FEV_1_ < 30%) (*n* = 40). In addition to airflow limitation severity, data on symptoms severity and history of exacerbations were used to differentiate COPD patients into GOLD groups based on COPD Assessment Test (CAT). There were GOLD A (*n* = 27), GOLD B (*n* = 70), GOLD C (*n* = 0) and GOLD D (*n* = 40) groups.

The control subjects were age- and sex-matched with the COPD subjects, and their medical history data and spirometry results were used to determine their health status. Subjects in both groups were older than 40 years, had no lung diseases (except COPD in COPD patients), inflammatory systemic diseases, acute infections, diabetes with severe complications, severe liver disease, severe renal insufficiency, malignant diseases, transplantations, or other specific or non-specific acute inflammation. In addition, all participants provided information on smoking status, so that there were 48 healthy non-smokers, 47 healthy smokers, 10 COPD non-smokers, 90 COPD former smokers, and 37 COPD smokers.

### 2.2. RNA Isolation and Gene Expression Analysis

Blood samples were collected in two tubes containing ethylenediaminetetraacetic acid (EDTA) anticoagulant (Greiner Bio-One, GmbH, Kremsmünster, Austria). One tube was used for DNA isolation, while the other was used for RNA extraction. After centrifugation at 3500 rpm for 10 min at +4 °C, RNA was extracted from buffy coat using the TRIzol/chloroform method [26], followed by the cDNA synthesis using RevertAid First Strand cDNA Synthesis Kit (Thermo Fischer Scientific, Waltham, MA, USA) with the GeneAmp PCR System 9700 (Applied Biosystems, Foster City, CA, USA) [27]. Commercial TaqMan Gene Expression Assays were used for the assessment of gene expression (Hs00359163_s1 for *HSP70*, Hs02621280_s1 for *TLR2*, Hs00152939_m1 for *TLR4*; Applied Biosystems, Foster City, CA, USA) as well as Taqman Universal Master Mix (Applied Biosystems, Foster City, CA, USA), according to the manufacturer’s guidelines. Beta-2-microglobulin (*B2M*) and peptidylprolyl isomerase (*PPIA*) ((Hs99999907_m1 for *B2M*, Hs99999904_m1for *PPIA*; Applied Biosystems, Foster City, CA, USA) were used as the endogenous controls for data normalization. The 2^−∆∆Ct^ method was used to calculate the relative expression of target genes in controls and COPD patients [28]. One randomly chosen control sample was included in each plate as a calibrator.

### 2.3. Determination of eHsp70 Concentration

Measurement of eHsp70 was determined in the same cohort in our previous study (8) in EDTA plasma using the commercially available enzyme-linked immunosorbent assay (ELISA) kit (Enzo Life Science, Farmingdale, NY, USA).

### 2.4. DNA Isolation and SNP Genotyping

DNA was extracted from blood cells by a standard salting-out procedure [29]. SNPs in the *HSP70*, *TLR2* and *TLR4* genes were selected based on a literature search of studies associated with COPD, smoking or other respiratory diseases, and when the minor allele frequency was (MAF) ≥0.05 in the European population, according to the 1000 Genomes Project. A total of nine polymorphisms shown in Table 1 were selected for genotyping by the Taqman allelic discrimination real-time PCR. Reaction was carried out in a total volume of 10 µL with the corresponding SNP Genotyping Assays (Applied Biosystems, Waltham MA, USA), TaqMan Genotyping Master Mix (Applied Biosystems, Foster City, CA, USA), and an adjusted volume of DNA templates, according to the manufacturer’s instructions. Analysis was performed in a 7500 Real-Time PCR System (Applied Biosystems, Foster City, CA, USA) using a default PCR amplification protocol. The successful call rate was greater than 96%, while 10% of samples were genotyped additionally to assess the reproducibility of the genotyping protocol. Data were interpreted using the Sequence Detection Software (SDS v. 1.4., Applied Biosystems, Waltham, MA, USA).

### 2.5. Statistics

Data were analyzed using MedCalc statistical software version 17.9.2. (MedCalc Software, Ostend, Belgium). All data failed a normality test performed with the Kolmogorov-Smirnov test, so a nonparametric Mann-Whitney test was used to assess the differences in age, spirometry parameters and the relative expression of target genes between the controls and COPD subjects. The Kruskal-Wallis test was used when more than two groups were compared. χ^2^ test was used to compare the categorical variable (sex), while the χ^2^ and Fisher’s exact test were used to test the relation between genotype and allele distribution. Logistic regression was performed for an association analysis between genotype and allele distribution, and COPD risk by calculating the odds ratio (OR) and the corresponding 95% confidence interval (CI).

The SNPStats software (https://www.snpstats.net/start.htm, accessed on 15 October 2020) was used for the assessment of Hardy-Weinberg equilibrium (HWE), linkage disequilibrium (LD), and haplotype analysis. A strong LD was considered when D’ > 0.80 [30].

The *p* value of <0.05 was considered statistically significant in all analyses.

## 3. Results

### 3.1. Basic Population Characteristics

The general information and spirometry results of the enrolled participants are shown in Table 2. COPD patients had decreased values of spirometry parameters in comparison to the controls. There was no difference in age or sex distribution between the two groups.

Age is shown as a median (minimum and maximum), while sex is shown as an absolute number. Other results are presented as a median (interquartile range). Data were analyzed by the χ^2^ or Mann-Whitney test.

### 3.2. HSP70, TLR2 and TLR4 Gene Expression in Controls and COPD Patients

The gene expression of *HSP70*, *TLR2* and *TLR4* was determined in peripheral blood cells, and it was found that the mRNA level was increased for *HSP70* and *TLR2* in patients with COPD, when compared to healthy subjects (Table 3).

Regarding the severity of airway obstruction as well as symptoms burden and the history of exacerbations (assessed by CAT), the gene expression of *HSP70* and *TLR2* was up-regulated in GOLD 2, GOLD 3 and GOLD 4 stages as well as in GOLD A, GOLD B and GOLD D groups, respectively, in comparison to the control group (Table 4). However, no significant differences between GOLD stages or groups were found.

*HSP70* gene expression showed to be increased in COPD non-smokers, COPD former smokers and COPD smokers in comparison to healthy non-smokers (Table 5). On the other hand, there was no difference between COPD non-smokers and healthy smokers, while the *HSP70* mRNA level was increased in COPD former and current smokers compared to healthy smokers. However, *HSP70* expression amongst COPD patients was independent of their smoking history. *TLR2* expression was increased in former COPD smokers and COPD smokers in comparison to healthy non-smokers, and only in COPD smokers when compared to healthy smokers. On the other hand, gene expression of *TLR4* did not differ between controls and COPD patients with different smoking history.

### 3.3. Association Analysis between HSP70, TLR2 and TLR4 SNPs and COPD

A total of nine SNPs of the *HSP70*, *TLR2* and *TLR4* genes were examined, and all SNPs had a call rate >96%. The genotype frequency distributions were consistent with HWE (*p* > 0.05), except for the *TLR2* rs1898830 SNP, so this variant was not included in further statistical analysis. Genotype and allele distribution of the studied polymorphisms was determined, and an association analysis was performed. It was found that the *HSP70* rs6457452 variant was associated with COPD risk when C/T and T/T carriers (OR = 0.48, 95% CI = 0.24–0.97, *p* = 0.040) were compared to C/C carriers (Table 6). In addition, there was a significant difference in allele distribution, and the T allele showed OR of 0.46 (95% CI = 0.24–0.89, *p* = 0.022) in comparison to the reference C allele. However, no significant relation between *TLR2* and *TLR4* polymorphisms and the risk of COPD was found (Table 6).

The associations between gene expression and polymorphisms of each gene were then examined. *HSP70* expression was increased in COPD C/C carriers of the rs6457452 polymorphism compared to COPD C/T carriers as well as in comparison to control C/C carriers (Table 7). On the other hand, relative gene expression of *HSP70* did not differ in the carriers of the C/T genotype when controls and COPD patients were compared. Reference homozygotes and heterozygotes of rs1008438 and rs1043618 who were COPD patients showed increased *HSP70* gene expression, while there was no difference in *HSP70* expression between control subjects and the COPD patient group within the variant homozygotes. The same was observed regarding the *TLR2* rs3804099 polymorphism, while the COPD carriers of the G/G genotype of the rs13150331 showed increased *TLR2* expression in comparison to healthy participants with the same genotype (Table S1). Finally, since *TLR4* expression did not differ between controls and COPD patients, there were no significant observations when *TLR4* expression was analyzed with respect to the three *TLR4* SNPs in all participants (Table S2).

Our previous research demonstrated that COPD patients had increased concentrations of plasma eHsp70 [8], and TLR2 and TLR4 are its main receptors [10]. Therefore, we evaluated the association between *HSP70*, *TLR2* and *TLR4* polymorphisms and eHsp70 concentrations (Table S3). COPD patients had increased eHsp70 concentrations compared to controls within the same genotype, while there was no difference in eHsp70 concentrations either in controls or COPD patients when heterozygotes and variant homozygotes were compared to reference homozygotes in terms of each polymorphism.

### 3.4. Haplotype Analysis

All three investigated *HSP70* SNPs were in strong LD, meaning that they are expected to be inherited together and therefore were included in the haplotype analysis. Table 8 shows that the C_rs1008438_C_rs1043618_T_rs6457452_ haplotype was associated with COPD risk with OR of 0.47 (95% CI = 0.23–0.97, *p* = 0.043) in comparison to the reference haplotype A_rs1008438_G_rs1043618_C_rs6457452_. Two *TLR2* polymorphisms were included in LD analysis. However, the D’ value between rs3804099 and rs13150331 was 0.65, so no haplotype analysis was performed due to the applied criterion of D’ > 0.80. Of the three *TLR4* SNPs examined, rs2737190 and rs10759932 as well as rs2737190 and rs7846989 were found to be strongly inherited. However, none of the common haplotypes with a frequency >0.01 was associated with COPD risk (Table S4).

## 4. Discussion

This study showed that the gene expression of *HSP70* and *TLR2* was increased in COPD patients compared to controls. This increase was independent of COPD severity assessed by both airway obstruction as well as symptoms burden and exacerbation history, and was also independent of the subjects’ smoking status. Regarding SNPs analysis, the T allele of *HSP70* rs6457452 polymorphism as well as *HSP70* haplotype consisting of the rs6457452 T allele had a protective role in COPD. Finally, decreased *HSP70* gene expression was observed in COPD C/T carriers of the rs6457452 polymorphism compared to COPD C/C homozygotes.

Dong et al. demonstrated increased *HSP70* gene expression in the peripheral lung tissues of COPD patients, and the *HSP70* expression negatively correlated with lung function which was assessed by FEV_1_. Moreover, it was observed that expression of Hsp70 protein and its mRNA level was up-regulated by cigarette smoke in vitro in 16HBE human bronchial epithelial cells [12]. In our study, gene expression of *HSP70* was increased in peripheral blood cells of COPD patients compared to the controls. We found that HSP70 gene expression amongst COPD patients was independent of their smoking history, and we suggest that observed increase of its level compared to controls was associated with disease itself and not with smoking. However, those conclusions should be taken with caution due to the small number of COPD non-smokers (*n* = 10). In the study by Rumora et al., Hsp70 protein expression was explored in relation to smoking status of both control and COPD subjects. Decreased Hsp70 expression in COPD individuals, especially in COPD smokers, was explained by its potentially increased release from peripheral blood leukocytes [31]. Indeed, we have previously reported increased plasma concentration of eHsp70 in COPD patients compared to healthy individuals [8].

The main eHsp70 receptors TLR2 and TLR4 are activated mostly by pathogen components but can also be activated by proteins released from dying cells, including Hsps that act like DAMPs. Activation of the TLR signaling pathway leads to the production of proinflammatory cytokines [32]. Overall, available data on *TLR2* and *TLR4* expression are conflicting, and results seem to be by cell type i.e., tissue specific. High gene expression of *TLR2* and *TLR4* was found in peripheral blood mononuclear cells of obese patients suggesting that up-regulated expression of *TLR*s is associated with persistent low-grade inflammation [33]. Both *TLR2* and *TLR4* mRNA levels were increased in circulating neutrophils of COPD patients, and they were also increased in COPD smokers compared to non-smoking controls [34]. Our previous in vitro studies demonstrated that eHsp70 increased only *TLR2* but not *TLR4* expression in human monocyte-derived macrophages [35]. Similarly, in the current study, *TLR2* gene expression was increased in peripheral blood cells of COPD patients, but *TLR4* expression in COPD subjects did not differ from the control group. This increase might be associated with increased levels of eHsp70. However, TLR2 is a receptor of many other ligands apart from eHsp70, and further investigation is needed to explore a possible association between TLR2 and eHsp70. *TLR2* showed similar pattern of behavior as *HSP70* in terms of association with disease severity but not entirely with smoking. *TLR2* expression increased only in former COPD smokers and COPD smokers compared with non-smoking controls, and in COPD smokers in comparison to smoking controls. It could be suggested that in stable COPD, especially in COPD smokers, there is a persistent low-grade inflammation that contributes to the increase of *HSP70* and *TLR2* mRNA levels. In addition, there may be differential transcription of the *HSP70* and *TLR2* genes in COPD patients, which could finally lead to differential gene expression.

A growing number of large-scale genome-wide association studies, e.g., COPD Gene and International COPD Genetics Consortium, have been trying to identify genetic variants associated with the risk of COPD that might help the diagnosis or become therapeutic targets. However, the heritability of complex diseases, such as COPD, remained unsolved. In order to explore the risk of developing COPD in the Croatian population, a total of nine SNPs of *HSP70*, *TLR2* and *TLR4* were genotyped. Only the relatively rare (MAF = 0.08) *HSP70* rs6457452 SNP, located in the 5′UTR region, showed an association with the risk of COPD. SNPs localized in regulatory part of a gene such as the 5′UTR region could affect the mRNA stability and expression, contributing to a disease susceptibility [36]. It was reported that rs6457452 was associated with increased risk of COPD in smokers, but the rs6457452 did not follow HWE in that study [13]. However, rs6457453 met the HWE criterion in our study and demonstrated a lower risk of COPD in the C/T and T/T carriers compared to C/C carriers, and the T allele showed protective effect. In the study by Guo et al. the T allele of rs6457452 was associated with an increased risk of lung cancer in two independent lung case-control studies within the Chinese population, and they demonstrated that the T allele was associated with lower Hsp70 protein expression in both normal bronchial epithelial cells and malignant cancer cells [37]. In addition, the C allele of rs6457452 was associated with decreased *HSP70* expression in sepsis. The same authors also estimated that the peripheral blood mononuclear cells of individuals with the haplotype consisting of the C allele of rs6457452 were producing less Hsp70 [38]. Haplotype analysis of *HSP70* from this study suggested an association of the haplotype containing the T allele of rs6457452 variant with the protective effect in COPD. When dealing with complex diseases, haplotype analysis is a useful and inexpensive method. Therefore, larger studies with a more detailed, statistically sophisticated assessment needs to be performed to explore further the effect of Hsp70 on the risk of COPD.

Although other polymorphisms studied did not show significant association with COPD risk in our cohort of participants, there were few studies that demonstrated their significant relations. The *HSP70* rs1008438 polymorphism was associated with COPD in all COPD subjects and in the smoking subgroup, and smoking parameter pack-years was higher in the COPD C/C carriers in the study by Korytina et al. [3]. In addition, it was reported that the A allele of rs1008438 was associated with increased COPD risk in smoking participants and those who were exposed to biomass burning smoke, but rs1008438 was not related to mRNA or protein levels of Hsp70 [13]. The C/C carriers of the *HSP70* rs1043618 were associated with an increased risk of lung cancer [39], and with higher risk of coronary heart disease, while reduced expression of Hsp70 protein was affected by the polymorphism [21]. *TLR2* and *TLR4* polymorphisms were associated with various conditions such as asthma [40], pulmonary tuberculosis [32,41], Helicobacter pylori infection [42], as well as alterations in levels of high-density lipoprotein cholesterol, low-density lipoprotein cholesterol and triglycerides [36]. They were also associated with susceptibility to COPD [25], the level and decline of lung function, an increased number of inflammatory cells in induced sputum of COPD patients [24], and an increased risk of COPD in smokers [43] or an earlier stage of COPD [44]. However, the ethnic context of polymorphisms as well as sample size should be considered regarding the results of the genetic association studies.

In this study, we found no association between plasma eHsp70 concentrations in COPD patients and selected *HSP70*, *TLR2* and *TLR4* polymorphisms. However, *HSP70* gene expression was affected by the *HSP70* rs6457452 SNP, and COPD patients with C/T genotype had reduced gene expression of *HSP70* in comparison to those with the C/C genotype. Furthermore, there was no difference in *HSP70* expression between the controls and COPD subjects within the same C/T genotype, suggesting that the T allele of the rs6457452 polymorphism may have an impact on gene transcription. However, this remains to be confirmed with a larger sample size that would include the COPD patients with T/T genotype, and the role of rs6457452 in COPD should be explored in a functional genomic study.

The main limitation of the current study is a small sample size, especially our results regarding the effect of smoking on *HSP70* and *TLR2* expression, and the association of *HSP70* rs6457452 with COPD and *HSP70* expression should be taken with caution. Another limitation of our study was the lack of GOLD 1 and GOLD C patients. However, COPD patients in GOLD 1 stage rarely contact physicians due to very mild symptoms, while GOLD C group of patients is also very rare since patients do not have many symptoms and are not frequent exacerbators.

## 5. Conclusions

To conclude, *HSP70* and *TLR2* gene expression may have a significant role in the pathogenesis of COPD. The current study is the first to demonstrate that *HSP70* rs6457452 polymorphism may affect *HSP70* gene expression and may be associated with the risk of COPD in the Croatian population. Thus, identification of specific molecular markers in peripheral circulation of COPD patients could be useful for achieving a better understanding of the systemic component of the disease.

## Figures and Tables

**Table 1:** Basic information about investigated polymorphisms in *HSP70*, *TLR2* and *TLR4* genes.

Gene Name	Gene ID	SNP ID	Assay ID	Functional Relevance	Nucleotide Substitution	Chromosome Position	MAF
*HSPA1A*	3303	rs1008438	ANCFDUH *	2 KB upstream variant	A > C	chr6:31815431	0.4036 (C)
*HSPA1A*	3303	rs1043618	C__11917510_10	5′UTR variant	G > C	chr6:31815730	0.3926 (C)
*HSPA1B*	3304	rs6457452	C___3052604_10	5′UTR variant	C > T	chr6:31827773	0.0775 (T)
*TLR2*	7097	rs1898830	C__11853988_10	intron variant	A > G	chr4:153687301	0.3250 (G)
*TLR2*	7097	rs3804099	C__22274563_10	synonymous variant	T > C	chr4:153703504	0.4354 (C)
*TLR2*	7097	rs13150331	C__11853987_10	intron variant	A > G	chr4:153678470	0.4056 (G)
*TLR4*	7099	rs2737190	C___2704047_10	upstream variant	G > A	chr9:117701903	0.3290 (G)
*TLR4*	7099	rs10759932	C__31783996_10	2 KB upstream variant	T > C	chr9:117702866	0.1531 (C)
*TLR4*	7099	rs7846989	C_189478937_10	3′UTR variant	T > C	chr9:117720662	0.1004 (C)

HSP—heat shock protein; TLR—Toll like receptor; SNP—single nucleotide polymorphism; MAF—minor allele frequency (1000 Genomes, European population); UTR—untranslated. * Custom Taqman^(R)^ SNP Genotyping Assay.

**Table 2:** Basic characteristics and spirometry parameters of healthy controls and COPD patients included in the study.

Parameter	Controls (*n* = 95)	COPD Patients (*n* = 137)	*p*
sex			0.118
male	49	86	
female	46	51	
age	64 (46–83)	65 (44–86)	0.073
FEV_1_ (L)	2.60 (2.12–3.19)	1.08 (0.78–1.57)	<0.001
FEV_1_ (% predicted)	93 (86–104)	39 (28–60)	<0.001
FVC (L)	3.35 (2.77–4.16)	2.28 (1.81–2.77)	<0.001
FEV_1_/FVC (%)	81 (77–88)	48 (41–58)	<0.001

COPD—chronic obstructive pulmonary disease; FEV_1_—forced expiratory volume in the first second; FVC—forced vital capacity.

**Table 3:** Relative expression of *HSP70*, *TLR2* and *TLR4* in controls and COPD patients.

	Controls	COPD Patients	*p*
*HSP70*	1.01 (0.74–2.07)	1.73 (1.11–7.46)	<0.001
*TLR2*	1.03 (0.69–2.65)	1.93 (0.95–6.10)	0.002
*TLR4*	0.72 (0.56–0.94)	0.77 (0.56–1.07)	0.323

HSP70—heat shock protein 70; TLR—Toll like receptor; COPD—chronic obstructive pulmonary disease. Data were analyzed by Mann-Whitney test and shown as a median (interquartile range).

**Table 4:** Relative expression of *HSP70*, *TLR2* and *TLR4* in controls and COPD patients according to the severity of airflow obstruction and symptoms severity.

	Controls (n = 95)	GOLD 2 (n = 47)	GOLD 3 (n = 50)	GOLD 4 (n = 40)	*p* ^1^	GOLD A (n = 27)	GOLD B (n = 70)	GOLD D (n = 40)	*p* ^2^
HSP70	1.01 (0.74–2.07)	1.67 (1.00–10.21)	1.92 (1.22–12.09)	1.68 (1.06–5.50)	<0.001	1.72 (1.12–11.61)	1.67 (1.09–6.04)	2.79 (1.17–7.46)	<0.001
TLR2	1.03 (0.69–2.65)	1.83 (1.00–3.67)	1.96 (1.02–10.64)	1.83 (0.90–6.92)	0.019	1.89 (1.18–5.26)	1.63 (0.82–3.81)	2.11 (1.00–8.51)	0.009
TLR4	0.72 (0.56–0.94)	0.78 (0.61–1.07)	0.80 (0.56–1.09)	0.71 (0.56–0.99)	0.690	0.76 (0.59–1.06)	0.76 (0.56–1.09)	0.80 (0.56–0.98)	0.797

HSP70—heat shock protein 70; TLR—Toll like receptor; COPD—chronic obstructive pulmonary disease; GOLD—Global Initiative for Chronic Obstructive Pulmonary Disease. Results are shown as a median (interquartile range). *p* ^1^—comparison between controls and GOLD 2–4 by Kruskal-Wallis test; *p* ^2^—comparison between controls and GOLD A-D by Kruskal-Wallis test.

**Table 5:** Relative expression of *HSP70*, *TLR2* and *TLR4* in controls and COPD patients based on smoking status.

	Healthy Non-Smokers (*n* = 48)	Healthy Smokers (*n* = 47)	COPD Non-Smokers (*n* = 10)	COPD Former Smokers (*n* = 90)	COPD Smokers (*n* = 37)	*p*
*HSP70*	0.93 (0.64–1.78)	1.09 (0.90–2.07)	1.87 ^a^ (1.49–2.11)	1.72 ^a,b^ (1.05–6.53)	2.14 ^a, b^ (1.21–12.93)	<0.001
*TLR2*	0.98 (0.70–3.08)	1.18 (0.65–2.65)	2.22 (1.17–3.94)	1.81 ^a^ (0.86–6.34)	1.98 ^a,b^ (1.32–12.59)	0.022
*TLR4*	0.65 (0.54–0.93)	0.78 (0.63–0.96)	0.78 (0.61–1.07)	0.80 (0.56–1.09)	0.71 (0.56–0.99)	0.482

HSP70—heat shock protein 70; TLR—Toll like receptor; COPD—chronic obstructive pulmonary disease. Data were tested by Kruskal-Wallis test and shown as median (interquartile range). ^a^ statistically significant difference in comparison to healthy non-smokers; ^b^ statistically significant difference in comparison to healthy smokers.

**Table 6:** Genotype and allele distribution of *HSP70*, *TLR2* and *TLR4* polymorphisms in controls and COPD patients, and association analysis between investigated polymorphisms and COPD risk.

HSP70			Controls n (%)	COPDn (%)	*p* ^1^	OR (95% CI)	*p* ^2^
rs1008438	genotype	A/A	33 (35)	60 (44)	0.192	1.00	
		A/C	50 (54)	57 (41)		0.61 (0.34–1.08)	0.089
		C/C	10 (11)	20 (15)		0.97 (0.41–2.27)	0.943
	allele	A	116 (62)	177 (65)	0.697	1.00	0.625
		C	70 (38)	97 (35)		0.91 (0.62–1.34)	
rs1043618	genotype	G/G	33 (36)	61 (45)	0.315	1.00	
		C/G	46 (51)	55 (40)		0.65 (0.36–1.15)	0.139
		C/C	12 (13)	20 (15)		0.90 (0.39–2.07)	0.807
	allele	G	112 (62)	177 (65)	0.504	1.00	
		C	70 (38)	95 (35)		0.86 (0.58–1.27)	0.443
rs6457452	genotype	C/C	71 (77)	120 (88)	0.046	1.00	
		C/T	19 (21)	17 (12)		0.48 (0.24–0.97)	0.040
		T/T	2 (2)	0 (0)			
	allele	C	161 (88)	257 (94)	0.030	1.00	
		T	23 (12)	17 (6)		0.46 (0.24–0.89)	0.022
TLR2							
rs3804099	genotype	T/T	29 (32)	45 (33)	0.668	1.00	
		C/T	44 (48)	59 (43)		0.86 (0.47–1.59)	0.638
		C/C	18 (20)	33 (24)		1.18 (0.56–2.48)	0.659
	allele	T	102 (56)	149 (54)	0.800	1.00	0.726
		C	80 (44)	125 (46)		1.07 (0.73–1.56)	
rs13150331	genotype	A/A	27 (29)	48 (36)	0.547	1.00	
		A/G	48 (52)	60 (44)		0.73 (0.39–1.34)	0.310
		G/G	17 (19)	27 (20)		0.95 (0.44–2.06)	0.903
	allele	A	102 (55)	156 (58)	0.690	1.00	
		G	82 (45)	114 (42)		0.91 (0.62–1.33)	0.621
TLR4							
rs2737190	genotype	G/G	14 (15)	19 (14)	0.600	1.00	
		A/G	35 (38)	59 (45)		1.24 (0.55–2.78)	0.599
		A/A	43 (47)	54 (41)		0.92 (0.42–2.06)	0.849
	allele	G	63 (34)	97 (37)	0.657	1.00	
		A	121 (66)	167 (63)		0.90 (0.60–1.33)	0.586
rs10759932	genotype	T/T	65 (72)	95 (70)	0.881	1.00	0.764
		C/T	21 (23)	40 (30)		1.09 (0.61–1.98)	
		C/C	4 (5)	0 (0)			
	allele	T	151 (84)	230 (85)	0.810	1.00	
		C	29 (16)	40 (15)		0.91 (0.54–1.52)	0.709
rs7846989	genotype	T/T	71 (77)	111 (83)	0.301	1.00	0.241
		C/T	20 (22)	22 (17)		0.67 (0.34–1.31)	
		C/C	1 (1)	0 (0)			
	allele	T	162 (88)	244 (92)	0.257	1.00	
		C	22 (12)	22 (8)		0.66 (0.36–1.24)	0.198

HSP70—heat shock protein 70; TLR2—Toll like receptor 2; TLR4—Toll-like receptor 4; OR—odds ratio; COPD—chronic obstructive pulmonary disease; CI—confidence interval. Allele and genotype distributions are presented as absolute numbers with corresponding percentages. *p* ^1^—*p*-value were analyzed by χ^2^ test or Fisher’s exact test where appropriate; *p* ^2^—*p*-value were analyzed by logistic regression.

**Table 7:** Association of *HSP70* polymorphisms with *HSP70* expression.

SNP	Genotype	*HSP70* Expression		*p* ^1^
		Controls	COPD	
rs1008438	A/A	0.90 (0.72–1.35)	2.02 (1.10–6.84)	<0.001
	A/C	1.09 (0.76–3.03)	1.72 (1.17–12.59)	0.005
	C/C	1.01 (0.71–1.38)	1.38 (1.07–2.78)	0.099
	P^2^	0.360	0.147	
rs1043618	G/G	0.90 (0.71–1.25)	2.02 (1.18–6.84)	<0.001
	C/G	1.09 (0.79–3.92)	1.72 (1.16–12.76)	0.008
	C/C	1.13 (0.78–1.67)	1.38 (1.07–2.78)	0.276
	P^2^	0.178	0.127	
rs6457452	C/C	0.98 (0.73–1.87)	1.83 (1.16–10.17)	<0.001
	C/T	1.31 (0.93–1.97)	1.27 (0.86–3.79)	0.921
	T/T	3.26 (1.01–5.50)	-	-
	P^2^	0.336	0.047	

SNP—single nucleotide polymorphism; HSP70—heat shock protein 70; COPD—chronic obstructive pulmonary disease. Results are shown as median (interquartile range). *p* ^1^—difference in *HSP70* expression between control group and patients with COPD within the same genotype of *HSP70* polymorphisms, checked by Kruskal-Wallis test. *p* ^2^—difference in *HSP70* expression between genotypes of *HSP70* polymorphisms in control group and in patients with COPD, checked by Kruskal-Wallis test.

**Table 8:** Association analysis of *HSP70* haplotypes with COPD risk.

rs1008438	rs1043618	rs6457452	Frequency		OR (95% CI)	*p*
			Controls	COPD		
A	G	C	0.570	0.646	1.00	-
C	C	C	0.230	0.285	1.03 (0.66–1.60)	0.910
C	C	T	0.107	0.062	0.47 (0.23–0.97)	0.043
C	G	C	0.039	0.007	0.19 (0.04–1.00)	0.052
A	C	C	0.035	0	NA	1

HSP70—heat shock protein 70; COPD—chronic obstructive pulmonary disease; OR—odds ratio; CI—confidence interval; NA—not applicable. Rare haplotypes (A_rs1008438_C_rs1043618_T_rs6457452_, C_rs1008438_G_rs1043618_T_rs6457452_ and A_rs1008438_G_rs1043618_T_rs6457452_) with total frequency <0.01 were not included in the association analysis.

## Data Availability

The data presented in this study are available on request from the corresponding author.

## Supplementary Materials

The following are available online at https://www.mdpi.com/article/10.3390/diagnostics11081412/s1, Table S1: Association of *TLR2* polymorphisms with *TLR2* expression; Table S2: Association of *TLR4* polymorphisms with *TLR4* expression; Table S3: Association of HSP70, *TLR2* and *TLR4* polymorphisms with plasma Hsp70 concentrations; Table S4: Association analysis of *TLR4* haplotypes with COPD risk.
