# Supplementary material for: Increased HSP70 and TLR2 Gene Expression and Association of HSP70 rs6457452 Single Nucleotide Polymorphism with the Risk of Chronic Obstructive Pulmonary Disease in the Croatian Population
Source: Diagnostics (Basel). 2021 Aug 4;11(8):1412. doi: 10.3390/diagnostics11081412 (PMC8394658; doi:10.3390/diagnostics11081412)
Supplement: Supplementary file 1 [file diagnostics-11-01412-s001.zip › diagnostics-1312113-supplementary.pdf]

Title:

Increased *HSP70* and *TLR2* gene expression and association of *HSP70* rs6457452 single nucleotide polymorphism with the risk of chronic obstructive pulmonary disease in the Croatian population

Authors:

Iva Hlapčić[1], Marija Grdić Rajković[1], Andrea Čeri[1], Sanja Dabelić[2], Sanja Popović-Grle[3,4], Margareta Radić Antolic[5], Jelena Knežević[6,7], Asta Försti[8,9], Lada Rumora[1]

[1]University of Zagreb, Faculty of Pharmacy and Biochemistry, Department of Medical Biochemistry and Haematology, Zagreb, Croatia

[2]University of Zagreb, Faculty of Pharmacy and Biochemistry, Department of Biochemistry and Molecular Biology, Zagreb, Croatia

[3]University Hospital Centre Zagreb, Clinical Department for Lung Diseases Jordanovac, Zagreb, Croatia

[4]University of Zagreb, School of Medicine, Zagreb, Croatia

[5]University Hospital Centre Zagreb, Clinical Institute of Laboratory Diagnostics, Zagreb, Croatia

[6]Ruđer Bošković Institute, Division of Molecular Medicine, Laboratory for Advanced Genomics, Zagreb, Croatia.

[7]Josip Juraj Strossmayer University of Osijek, Faculty for Dental Medicine and Health, Department of Integrative Medicine, Osijek, Zagreb

[8]German Cancer Research Center (DKFZ), German Cancer Consortium (DKTK), Division of Pediatric Neurooncology, Heidelberg, Germany

[9]Hopp Children’s Cancer Center (KiTZ), Heidelberg, Germany

*Corresponding author: Lada Rumora, full professor; University of Zagreb, Faculty of Pharmacy and Biochemistry, Department of Medical Biochemistry and Haematology; Kneza Domagoja 2, 10000 Zagreb, Croatia; Telephone: +38516394782, Fax: +38514612716, e-mail: lada.rumora@pharma.unizg.hr

Table S1. Association of *TLR2* polymorphisms with *TLR2* expression.

| SNP | genotype | *TLR2* expression | | P* |
|---|---|---|---|---|
| | | controls | COPD | |
| rs3804099 | T/T | 1.02 (0.64 – 2.25) | 2.07 (1.01 – 5.56) | **0.027** |
| | C/T | 0.99 (0.71 – 2.01) | 1.83 (0.93 – 10.17) | **0.026** |
| | C/C | 1.31 (0.80 – 7.24) | 1.97 (0.91 – 5.14) | 0.657 |
| | P** | 0.492 | 0.827 | |
| rs13150331 | A/A | 1.15 (0.74 – 1.70) | 1.98 (0.75 – 3.67) | 0.169 |
| | A/G | 1.16 (0.69 – 7.24) | 1.93 (0.94 – 7.73) | 0.132 |
| | G/G | 1.00 (0.66 – 1.26) | 1.78 (1.13 – 9.19) | **0.007** |
| | P** | 0.410 | 0.754 | |

SNP – single nucleotide polymorphism; TLR2 – Toll like receptor 2; COPD – chronic obstructive pulmonary disease.

Results are shown as a median (interquartile range).

P* - difference in *TLR2* expression between control group and patients with COPD within the same genotype of *TLR2* polymorphisms, checked by Kruskal-Wallis test.

P** - difference in *TLR2* expression between genotypes of *TLR2* polymorphisms in control group and in patients with COPD, checked by Kruskal-Wallis test.

Table S2. Association of *TLR4* polymorphisms with *TLR4* expression.

| SNP | genotype | *TLR4* expression controls | *TLR4* expression COPD | P* |
|---|---|---|---|---|
| rs2737190 | G/G | 0.84 (0.64 – 0.95) | 0.87 (0.60 – 1.26) | 0.687 |
| | A/G | 0.72 (0.59 – 0.89) | 0.75 (0.56 – 1.09) | 0.745 |
| | A/A | 0.65 (0.55 – 0.94) | 0.78 (0.55 – 0.96) | 0.371 |
| | P** | 0.347 | 0.470 | |
| rs10759932 | T/T | 0.68 (0.55 – 0.94) | 0.79 (0.56 – 1.03) | 0.288 |
| | T/C | 0.69 (0.54 – 0.86) | 0.71 (0.59 – 1.11) | 0.333 |
| | C/C | 0.90 (0.86 – 1.43) | - | - |
| | P** | 0.200 | 0.755 | |
| rs7846989 | T/T | 0.72 (0.55 – 0.94) | 0.76 (0.56 – 1.08) | 0.350 |
| | C/T | 0.69 (0.63 – 0.85) | 0.77 (0.61 – 0.98) | 0.583 |
| | C/C | 0.99 (0.99 – 0.99) | - | - |
| | P** | 0.541 | 0.693 | |

SNP – single nucleotide polymorphism; TLR4 – Toll like receptor 4; COPD – chronic obstructive pulmonary disease.

Results are shown as a median (interquartile range).

P* - difference in *TLR4* expression between control group and patients with COPD within the same genotype of *TLR4* polymorphisms, checked by Kruskal-Wallis test.

P** - difference in *TLR4* expression between genotypes of *TLR4* polymorphisms in control group and in patients with COPD, checked by Kruskal-Wallis test.

Table S3. Association of *HSP70*, *TLR2* and *TLR4* polymorphisms with plasma Hsp70 concentrations.

| gene | SNP | genotype | eHsp70 (ng/ml) | | P* |
|---|---|---|---|---|---|
| | | | controls | COPD | |
| *HSP70* | rs1008438 | A/A | 0.35 (0.24 – 0.48) | 0.99 (0.59 – 1.20) | <**0.001** |
| | | A/C | 0.40 (0.25 – 0.72) | 0.99 (0.70 – 1.31) | <**0.001** |
| | | C/C | 0.41 (0.25 – 0.82) | 0.93 (0.58 – 1.43) | **0.019** |
| | | P** | 0.316 | 0.841 | |
| | rs1043618 | G/G | 0.38 (0.26 – 0.62) | 0.97 (0.57 – 1.20) | <**0.001** |
| | | C/G | 0.39 (0.25 – 0.74) | 0.99 (0.74 – 1.31) | <**0.001** |
| | | C/C | 0.37 (0.26 – 0.63) | 0.93 (0.58 – 1.43) | **0.004** |
| | | P** | 0.920 | 0.605 | |
| | rs6457452 | C/C | 0.37 (0.25 – 0.60) | 0.99 (0.62 – 1.28) | <**0.001** |
| | | C/T | 0.37 (0.21 – 0.70) | 0.98 (0.66 – 1.31) | <**0.001** |
| | | T/T | 0.79 (0.37 – 1.22) | - | - |
| | | P** | 0.563 | 0.901 | |
| *TLR2* | rs3804099 | T/T | 0.37 (0.27 – 0.56) | 0.94 (0.57 – 1.24) | <**0.001** |
| | | C/T | 0.37 (0.25 – 0.67) | 1.06 (0.80 – 1.26) | <**0.001** |
| | | C/C | 0.34 (21 – 0.74) | 0.94 (0.65 – 1.32) | **0.001** |
| | | P** | 0.831 | 0.315 | |
| | rs13150331 | A/A | 0.38 (0.23 – 0.70) | 0.98 (0.57 – 1.30) | <**0.001** |
| | | A/G | 0.37 (0.27 – 0.88) | 0.99 (0.63 – 1.30) | <**0.001** |
| | | G/G | 0.31 (0.17 – 0.41) | 0.98 (0.71 – 1.18) | <**0.001** |
| | | P** | 0.252 | 0.972 | |

| | | | | | |
|---|---|---|---|---|---|
| *TLR4* | rs2737190 | G/G | 0.41 (0.26 – 0.50) | 0.98 (0.73 – 1.28) | **<0.001** |
| | | A/G | 0.47 (0.26 – 0.75) | 1.03 (0.59 – 1.29) | **<0.001** |
| | | A/A | 0.35 (0.22 – 0.48) | 0.96 (0.60 – 1.20) | **<0.001** |
| | | P** | 0.467 | 0.616 | |
| | rs10759932 | T/T | 0.37 (0.27 – 0.64) | 0.98 (0.60 – 1.33) | **<0.001** |
| | | T/C | 0.42 (0.16 – 0.76) | 0.98 (0.78 – 1.19) | **<0.001** |
| | | C/C | 0.31 (0.25 – 0.42) | - | - |
| | | P** | 0.784 | 0.931 | |
| | rs7846989 | T/T | 0.37 (0.23 – 0.64) | 0.98 (0.60 – 1.29) | **<0.001** |
| | | C/T | 0.40 (0.28 – 0.62) | 1.03 (0.75 – 2.14) | **<0.001** |
| | | C/C | 0.25 (0.25 – 0.25) | - | - |
| | | P** | 0.635 | 0.257 | |

HSP70 – heat shock protein 70; TLR – Toll like receptor; SNP – single nucleotide polymorphism; COPD – chronic obstructive pulmonary disease; eHsp70 – extracellular Hsp70.

Results are shown as a median (interquartile range).

P* - difference in eHsp70 concentration between control group and patients with COPD within the same genotype of *HSP70*, *TLR2* or *TLR4* polymorphisms, checked by Kruskal-Wallis test.

P** - difference in eHsp70 concentration between genotypes of *HSP70, TLR2* or *TLR4* polymorphisms in control group and in patients with COPD, checked by Kruskal-Wallis test.

Table S4. Association analysis of *TLR4* haplotypes with COPD risk.

| rs2737190 | rs10759932 | rs7846989 | frequency | | OR (95% CI) | P |
|---|---|---|---|---|---|---|
| | | | controls | COPD | | |
| A | T | T | 0.641 | 0.615 | 1.00 | - |
| G | T | T | 0.121 | 0.179 | 1.40 (0.83 – 2.35) | 0.210 |
| G | C | T | 0.111 | 0.113 | 1.09 (0.57 – 2.09) | 0.790 |
| G | T | C | 0.062 | 0.057 | 0.89 (0.38 – 2.10) | 0.800 |
| G | C | C | 0.051 | 0.019 | 0.39 (0.12 – 1.30) | 0.130 |

TLR4 – Toll like receptor 4; COPD – chronic obstructive pulmonary disease; OR – odds ratio; CI – confidence interval.

Rare haplotypes ($A_{rs2737190}C_{rs10759932}T_{rs7846989}$, $A_{rs2737190}T_{rs10759932}C_{rs7846989}$ and $A_{rs2737190}C_{rs10759932}C_{rs7846989}$) with total frequency <0.01 were not included in the association analysis.
